# Advances in Alzheimer’s disease’s pharmacological treatment

**DOI:** 10.3389/fphar.2023.1101452

**Published:** 2023-01-26

**Authors:** Carlos Elias Conti Filho, Lairane Bridi Loss, Clairton Marcolongo-Pereira, Joamyr Victor Rossoni Junior, Rafael Mazioli Barcelos, Orlando Chiarelli-Neto, Bruno Spalenza da Silva, Roberta Passamani Ambrosio, Fernanda Cristina de Abreu Quintela Castro, Sarah Fernandes Teixeira, Nathana Jamille Mezzomo

**Affiliations:** Faculty of Medicine, University Center of Espirito Santo, Colatina, Brazil

**Keywords:** Alzheimer’s disease, molecular target, drug development, pharmacological treatment, new drugs

## Abstract

Alzheimer’s disease (AD) is the most common type of dementia in the elderly. Several hypotheses emerged from AD pathophysiological mechanisms. However, no neuronal protective or regenerative drug is available nowadays. Researchers still work in drug development and are finding new molecular targets to treat AD. Therefore, this study aimed to summarize main advances in AD pharmacological therapy. Clinical trials registered in the National Library of Medicine database were selected and analyzed accordingly to molecular targets, therapeutic effects, and safety profile. The most common outcome was the lack of efficacy. Only seven trials concluded that tested drugs were safe and induced any kind of therapeutic improvement. Three works showed therapeutic effects followed by toxicity. In addition to aducanumab recent FDA approval, antibodies against amyloid-β (Aβ) showed no noteworthy results. 5-HT6 antagonists, tau inhibitors and nicotinic agonists’ data were discouraging. However, anti-Aβ vaccine, BACE inhibitor and anti-neuroinflammation drugs showed promising results.

## Introduction

It is estimated that 55 million people have dementia worldwide and, by 2050, this number may increase to 139 million due to population aging. In 2019, dementia global cost was estimated to be 1.3 trillion dollars and led to 1.6 million deaths (WHO, 2017; WHO, 2021; WHO, 2022). Alzheimer’s disease (AD) is the most common type of dementia in the elderly and affects mainly females. It was estimated that among AD diagnosis, 44% are 75- to 84-year-old patients and 38% are 85 years or older. Thus, AD is a social and economic global burden (Hebert et al., 2013). This neurodegenerative disease is related to loss of cognitive functions caused by several pathological pathways: amyloid-β (Aβ) deposition, hyperphosphorylated tau protein, cholinergic disorder, excessive glutamatergic stimulation, oxidative stress, and neuroinflammation (Hardy and Allsop, 1991; Gomez-Isla et al., 1997; Fiala et al., 2007; Holmes et al., 2009; Tolar et al., 2020). The first case was reported by Alois Alzheimer in 1906, and, despite all improvements in understanding AD pathogenesis, nowadays, therapies only help to manage some symptoms. AD lingers without a cure or strategy to mitigate its progression (Alzheimer, 1906; Alzheimer et al., 1995; Tolar et al., 2020).

AD diagnosis continues to be mainly based on clinical evaluation of cognitive and physical examination. However, pathological changes occur years before symptoms arise, and the earlier diagnosis may be accomplished by detecting molecular biomarkers (Aβ and tau) or cortical atrophy using magnetic resonance imaging. Despite all available technology, the greatest sensitivity and efficacy is found only in *postmortem* cerebral autopsy (Ranasinghe et al., 2021; Vaillant-Beuchot et al., 2021; Troutwine et al., 2022).

Current treatments available include use of cholinesterase inhibitors for patients with any stage of AD and memantine for people with moderate to severe dementia. Main drugs approved are rivastigmine, galantamine, memantine and donepezil. However, they only improve quality of life when prescribed at the appropriate time (Botchway et al., 2018; Scheltens et al., 2021). Two decades after memantine approval, the United States (US) Food and Drug Administration (FDA) approved aducanumab in 2021, the first monoclonal antibody anti-Aβ and the latest AD drug approved. In addition to the entire thrill, this new drug is expensive and there is some doubt related to its benefits (Mafi et al., 2022). Therefore, this study aimed to describe main advances in AD pharmacological therapy through an analysis of latest clinical trial results registered in the US National Institutes of Health (NIH), contributing to theoretical information for drug development pipelines and future clinical practice.

## Potential targets for drug design

**FIGURE 1:**
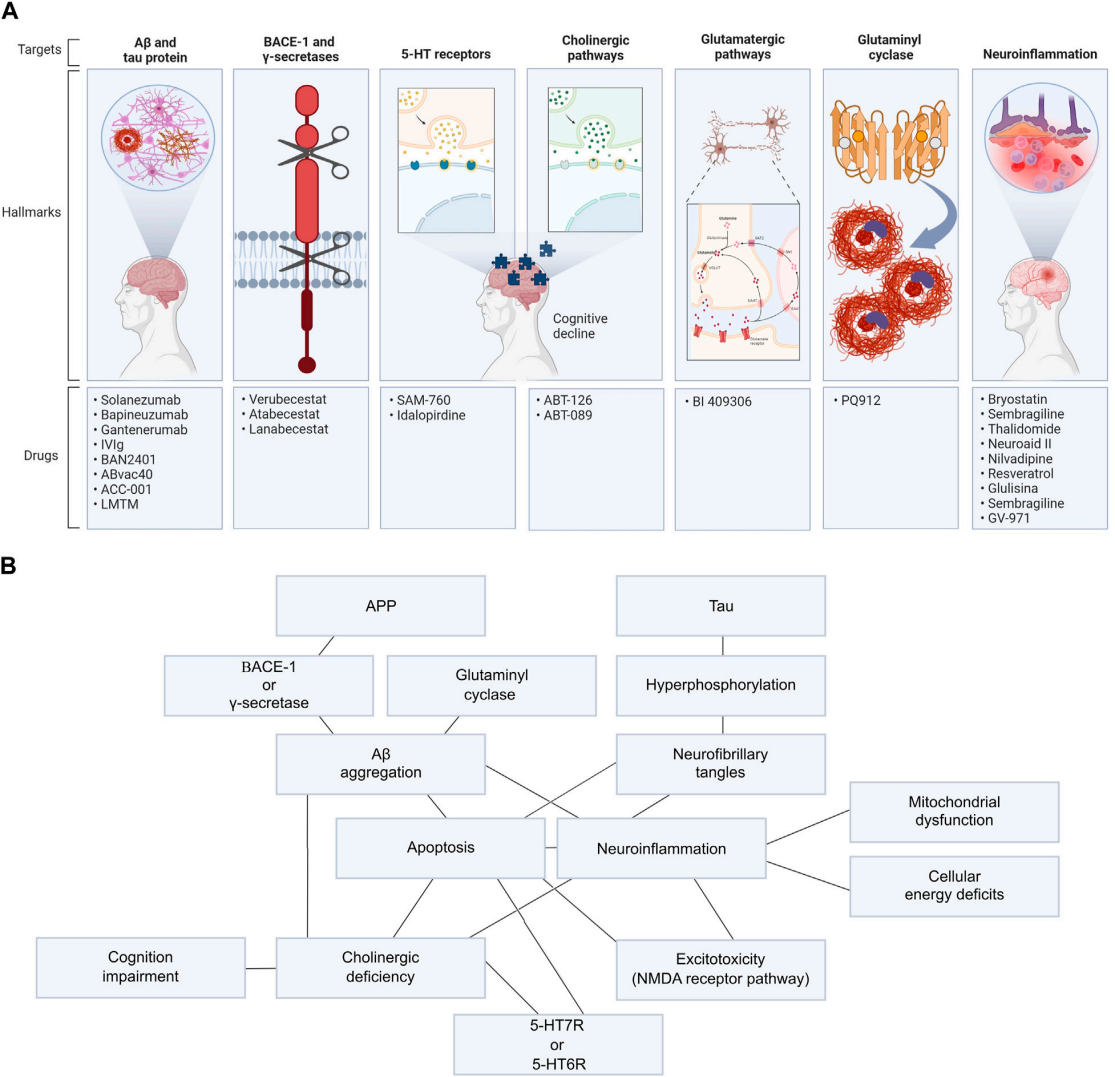
Main potential targets for drug design in AD and relation of its pathways. **(A)** AD’s targets used to develop new drugs in clinical trials evaluated in this work. **(B)** All mechanisms related to AD pathogenesis and progression are connected and were explored in these clinical trials, such as Aβ and tau aggregation; BACE-1, γ-secretase, or glutaminyl cyclase activity; neuroinflammation; excitotoxicity; 5-HT7R or 5-HT6R hyperstimulation and cholinergic impairment.

This work analyzed 43 AD new drug clinical trials registered in the National Library of Medicine database ClinicalTrials.gov funded by the NIH with data published in PubMed between 2015 and October 2022, using the following keywords: “Alzheimer’s disease” AND “drug.” Molecular target, therapeutic effect, safety profile and side effects were evaluated in each study (Table 1). Only new drugs’ clinical trials registered in the NIH were included. Exclusion criteria were as follows: drugs already approved by the FDA (even if it is in a new formulation or delivery system) and pharmacological strategies that aim to solve only non-cognitive or degenerative symptoms. Those works tested 27 new drugs and 23 different molecular targets. In summary, we evaluated three anti-Aβ therapeutic vaccines, five anti-Aβ antibodies, a tau aggregation inhibitor, three BACE-1 inhibitors, 2 5-HT6 receptor antagonists, 2 nicotinic receptors agonists, a muscarinic agonist, a glutaminyl cyclase inhibitor and 10 anti-neuroinflammation drugs (Figure 1). Pathways related to those drugs are better detailed in Supplementary Data S1.

**TABLE 1:** Information of clinical trials reviewed.

Reference	Drug	Trial phase	Therapeutic target	Patients	Clinical trial number	Therapeutic effects	Safety	Side effects
Lenz et al. (2015)	ABT-089	2	α4β2 neuronal nicotinic receptor	Mild to moderate AD	NCT00555204	Lack of efficacy	Safe and well-tolerated	No data available
Gault et al. (2016), Florian et al. (2016)	ABT-126 (nelonicline)	2	α7 nicotinic receptor	Mild to moderate AD	NCT01527916; NCT01549834; NCT01676935	Lack of efficacy	Safe and well-tolerated	Agitation, constipation, diarrhea, fall and headache
Lacosta et al. (2018)	ABvac40	1	C-terminal of Aβ40	Mild to moderate AD	NCT03113812	Anti-Aβ40 antibodies production	Safe and well-tolerated	No data available
Timmers et al. (2018)	Atabecestat	1	BACE-1	Mild AD	NCT01978548; NCT02360657	Aβ reduction in CSF	Safe and well-tolerated	No data available
Logovinsky et al. (2016)	BAN2401	1	Aβ protofibril	Mild to moderate AD	NCT01230853	Plasma Aβ1-40 increase	Safe and well-tolerated	No data available
Brody et al. (2016), Salloway et al. (2018)	Bapineuzumab	2	N-terminus of Aβ	Mild to moderate AD	NCT01254773; NCT00606476	Lack of efficacy	Safe and well-tolerated	Cataract, injection site hemorrhage, nasopharyngitis, pneumonia and muscle twitching
Vandenberghe et al. (2016), Ivanoiu et al. (2016) Salloway et al. (2018)	Bapineuzumab	3	N-terminus of Aβ	Mild to moderate AD and prodromal AD	NCT00667810; NCT00996918; NCT00676143; NCT00937352; NCT00998764	Lack of efficacy	Safe and well-tolerated	Cataract, injection site hemorrhage, nasopharyngitis, pneumonia and muscle twitching
Arai et al. (2016)	Bapineuzumab	1	N-terminus of Aβ	Mild to moderate AD	NCT00397891	No results	Safe and well-tolerated	No data available
Frölich et al. (2019)	BI 409306	2	Phosphodiesterase type 9	Mild AD	NCT02240693; NCT02337907	Lack of efficacy	Safe and well-tolerated	No data available
Farlow et al. (2019)	Bryostatin	2	PKC epsilon activator	Advanced AD	NTRP101-202	Cognitive improvement	Safe and well-tolerated in low doses	No data available
Butchart et al. (2015)	Etanercept	2	TNF-α	Mild to moderate AD	NCT01068353	Lack of efficacy	Safe and well-tolerated	No data available
Ostrowitzi et al. (2017)	Gantenerumab	3	N-terminal and central amino acids of Aβ	Prodromal AD	NCT01224106	Lack of efficacy	Safe and well-tolerated	No data available
Rosenbloom et al. (2021)	Glulisina	2	Insulin	Mild and prodromal AD	NCT02503501	Lack of efficacy	Safe and well-tolerated	No data available
Bakker et al. (2021)	HTL0018318	1	M1 receptor	Healthy elderly and adults	NCT03456349	No results	Safe and well-tolerated	No data available
Atri et al. (2018)	Idalopirdine	3	5-HT6 receptor	Mild to moderate AD	NCT01955161; NCT02006641; NCT02006654	Lack of efficacy	Safe and well-tolerated	No data available
Relkin et al. (2017)	Intravenous immunoglobulin (IVIg)	3	Aβ	Mild to moderate AD	NCT00818662	Lack of efficacy	Safe and well-tolerated	No data available
Sakamoto et al. (2017)	Lanabecestat	1	BACE-1	Mild AD	NCT02005211	Aβ reduction in CSF and plasma	Safe and well-tolerated	No data available
Gauthier et al. (2016), Wilcock et al. (2018)	Leuco-methylthioninium bis (LMTM)	3	Tau	Mild to moderate AD	NCT01689246; NCT01689233	Lack of efficacy	Toxicity	Diarrhea, dysuria and decreased hemoglobin
Chen et al. (2022)	MLC901 (Neuroaid II)	2	ATP-sensitive potassium channels	Mild to moderate AD	NCT03038035	Potential decrease of AD progression	Safe and well-tolerated	No data available
Lawlor et al. (2018)	Nilvadipine	3	Calcium channel blocker	Mild to moderate AD	NCT02017340	Lack of efficacy	Safe and well-tolerated	No data available
Scheltens et al. (2018)	PQ912	2	Glutaminyl cyclase	Mild AD	NCT02389413	EEG frequency decrease and cognitive improvement	Toxicity	Nausea, diarrhea, constipation, infections, rash and urticaria
Moussa et al. (2017), Turner et al. (2015)	Resveratrol	2	SIRT1	Mild to moderate AD	NCT01504854	Attenuation of cognitive and functional decline	Safe and well-tolerated	Nausea and diarrhea
Fullerton et al. (2018)	SAM-760	2	5-HT6 receptor	Mild to moderate AD	NCT01712074	Lack of efficacy	Safe and well-tolerated	No data available
Nave et al. (2017)	Sembragiline	2	MAO-B	Moderate AD	NCT01677754	Only neuropsychiatric symptoms improvement	Safe and well-tolerated	No data available
Xiao et al. (2021)	Sodium oligomannate (GV-971)	3	Gut microbiota	Mild to moderate AD	NCT04520412	Cognitive improvement	Safe and well-tolerated	No data available
Liu-Seifert et al. (2018), Honig et al. (2018)	Solanezumab	3	Mid-domain of Aβ	Mild to moderate AD	NCT01900665	Lack of efficacy	Safe and well-tolerated	No data available
Decourt et al. (2017)	Thalidomide	2	TNF-α	Mild to moderate AD	NCT01094340	Lack of efficacy	Toxicity	Reduction in brain volume and neurological, urinary, gastrointestinal and skin adverse events
Pasquier et al. (2016), Van Dyck et al. (2016)	Vanutide cridificar (ACC-001)	2	N-terminal of Aβ1-7	Mild to moderate AD	NCT00479557; NCT00498602; NCT01227564	Lack of efficacy	Safe and well-tolerated	No data available
Egan et al. (2019)	Verubecestat	2	BACE-1	Prodromal AD	NCT01953601	Cognition and daily function decrease	Toxicity	Rash-related events, hair-color changes, falls, injuries, weight loss, and neuropsychiatric symptoms
Egan et al. (2018)	Verubecestat	3	BACE-1	Mild to moderate AD	NCT01739348	Lack of efficacy	Toxicity	No data available

In summary, the main issue among failed trials was lack of efficiency (Butchart et al., 2015; Lenz et al., 2015; Brody et al., 2016; Florian et al., 2016; Gault et al., 2016; Gauthier et al., 2016; Ivanoiu et al., 2016; Pasquier et al., 2016; Van Dyck et al., 2016; Vandenberghe et al., 2016; Decourt et al., 2017; Ostrowitzki et al., 2017; Relkin et al., 2017; Atri et al., 2018; Egan et al., 2018; Fullerton et al., 2018; Honig et al., 2018; Lawlor et al., 2018; Liu-Seifert et al., 2018; Salloway et al., 2018; Wilcock et al., 2018; Frölich et al., 2019; Rosenbloom et al., 2021). Good outcomes were obtained in seven trials that induced any type of therapeutic improvement without toxicity (Turner et al., 2015; Logovinsky et al., 2016; Moussa et al., 2017; Nave et al., 2017; Sakamoto et al., 2017; Lacosta et al., 2018; Timmers et al., 2018; Xiao et al., 2021; Chen et al., 2022). Three works showed therapeutic effect followed by toxicity (Scheltens et al., 2018; Egan et al., 2019; Farlow et al., 2019).

### Aβ and tau protein

Main therapeutic targets studied for AD were directly or indirectly related to neurofibrillary tangles (tau protein) and senile plaques (Aβ protein). Nevertheless, only aducanumab, an antibody anti-Aβ, was approved (Tolar et al., 2020; Mafi et al., 2022). Aβ and tau proteins highlight as drug targets and are related to AD pathogenesis. Amyloid precursor protein (APP) cleavage by β-secretases (BACE-1) or γ-secretases results in insoluble Aβ protein, a hallmark of AD. Therefore, the therapeutic rationale is to disassemble and degrade amyloid plaques chemically or by recruiting microglia and activating phagocytosis to stop or undo neuronal damage triggered by those protein accumulation (Da Mesquita et al., 2021; Vaillant-Beuchot et al., 2021; Troutwine et al., 2022). Active or passive immunotherapies are the most studied strategies due to their specific response, although they may induce autoantibodies, edema or hemorrhage (Geylis and Steinitz, 2006; Sengupta et al., 2016). Indeed, those following trials might have failed due to the timing of intervention. Earlier treatments could lead to better results (Botchway et al., 2018; Scheltens et al., 2021).

Solanezumab (Honig et al., 2018; Liu-Seifert, et al., 2018), bapineuzumab (Arai et al., 2016; Brody et al., 2016; Ivanoiu et al., 2016; Vandenberghe et al., 2016; Salloway et al., 2018a; Salloway et al., 2018b), BAN2401 (Logovinsky et al., 2016), and gantenerumab (Ostrowitzki et al., 2017) are monoclonal anti-Aβ antibodies. In addition to bapineuzumab phase 1 trial results of increasing plasma concentrations of β-amyloid1-40 in patients (Arai et al., 2016), all anti-Aβ antibodies failed in therapeutic efficiency, discouraging further studies. Another strategy was developed from natural anti-Aβ antibodies arising in the absence of vaccination or passive immunization in adults’ blood or AD patients. Relkin et al. (2017) evaluated intravenous immunoglobulins IgG-type (IVIg) derived from plasma of healthy donors to treat patients with mild to moderate AD. In addition to a good safety profile and decreasing plasma Aβ42, no clinical effect was observed.

On the other hand, clinical studies of vaccines were less encouraged due to the full-length Aβ1–42 peptide (AN1792) trial safety issues. This work demonstrated decrease in Aβ plaques, benefits on some cognitive and memory measures, and reductions in cerebrospinal fluid (CSF) tau. However, meningoencephalitis was presented in approximately 6% of AN1792-treated patients due to cytotoxic T-cell response (Nicoll et al., 2003; Ferrer et al., 2004; Masliah et al., 2005). Safety is no longer an issue in recent studies, which included vanutide cridificar (ACC-001), a vaccine designed to elicit antibodies against N-terminal peptide Aβ1-7. In this work, safety and tolerability profile was acceptable, but it lacks therapeutic effect (Pasquier et al., 2016; Van Dyck et al., 2016). ABvac40, a vaccine against the C-terminal end of Aβ40, as well showed safety and tolerability, but also, most of the individuals receiving ABvac40 developed specific anti-Aβ40 antibodies (Lacosta et al., 2018).

In addition to tau central role in AD pathogenesis, clinical trials focusing on this target are rare because of toxicity and/or lack of efficacy. Tau provides microtubule stability and contributes to the regulation of intracellular trafficking by its phosphorylation. Hyperphosphorylation of tau destabilizes tau–microtubule interactions, leading to instability, transport defects along microtubules and neuronal death (Dixit et al., 2008; Iqbal et al., 2010; Vershinin et al., 2017). Methylthioninium chloride (MTC; commonly known as “methylene blue”) was the first reported tau aggregation inhibitor (TAI) without disrupting normal tau–tubulin interactions (Melis et al., 2015). However, MTC had no therapeutic effect in phase 2 trial probably due to its poor tolerability and pharmacokinetic issues. MTC exists in equilibrium with its oxygen-sensitive redox couple, leucomethylthionium (LMTM) (Harrington et al., 2015). Although LMTM combines superior pharmacological properties with TAI activity than MTC, phase 3 trial showed no benefit to AD patients and led to treatment discontinuation (Gauthier et al., 2016; Wilcock et al., 2018).

### BACE-1 and γ-secretases

Other important targets related to AD pathogenesis are BACE-1 and γ-secretases; once those enzyme activities were responsible for Aβ development, as described earlier in this work. However, disappointingly several inhibitors of those enzyme studies were discontinued due to futility and toxicity, including cognitive impairment. Severe toxicity was especially high in γ-secretase inhibitors, indicating that its inhibition cannot be achieved in a safe way due to its physiologic effect in the Notch pathway (Cebers et al., 2017; McDade et al., 2021; Yang et al., 2021).

Verubecestat, an oral BACE-1 inhibitor, had terrible clinical trial outcomes. Moreover, in phase 2, cognition and daily function decreased in patients using verubecestat and showed no therapeutic effect in phase 3 trial (Egan et al., 2018; Egan et al., 2019). On its turn, atabecestat is an oral BACE inhibitor capable of decreasing cerebrospinal fluid (CSF) Aβ1-40 with low toxicity (Timmers et al., 2018). Another oral BACE inhibitor is lanabecestat (AZD3293; LY3314814), and it decreased plasma and CSF Aβ (Sakamoto et al., 2017).

### 5-HT receptors

Lately, serotonin receptors emerged as targets in cognitive impairment and AD (Hung and Fu, 2017; Andrews et al., 2018; Kucwaj-Brysz et al., 2021; Solas et al., 2021; Higa et al., 2022). 5-HT6R and 5-HT7R are the most studied serotonin receptors in this scenario due to their brain distribution and noteworthy cognitive properties *in vivo* (Kucwaj-Brysz et al., 2021). Furthermore, 5-HT6R induces signaling that changes cholinergic, monoaminergic, and glutamatergic brain signaling with little periphery adverse effects (Andrews et al., 2018; Liu et al., 2019; Kucwaj-Brysz et al., 2021). However, no selective 5-HT6R or 5-HT7R drug successfully confirmed its therapeutic activity in clinical trials (Hung and Fu, 2017; Andrews et al., 2018; Kucwaj-Brysz et al., 2021). Main adverse effects related to those targets are decreased food intake and body weight (Andrews et al., 2018).

PF-05212377 (SAM-760) and idalopirdine are selective antagonists of 5-HT6R with a good safety profile; however, they failed to demonstrate efficacy. Altogether, those findings suggest that 5-HT6 antagonists should not be a main target to AD therapy (Atril et al., 2018; Fullerton et al., 2018).

### Cholinergic pathways

According to the cholinergic hypothesis, AD is related to the reduction of acetylcholine. Therefore, most frequent pharmacologic therapy for AD is to increase cholinergic pathways through acetylcholinesterase inhibition (IAch) (Bartus et al., 1982; Recio-Barbero et al., 2021). IAch drugs, such as rivastigmine, donepezil, tacrine and galantamine, only provide a modest and not lingering symptomatic benefit to cognitive decline (Mohammad et al., 2017; Kumar et al., 2020; Sabandal et al., 2022). Most common side effects in cholinergic drugs are gastrointestinal issues, fatigue, cramps and sinus node dysfunction (Briggs et al., 2016; Mohammad et al., 2017).

A novel cholinergic approach for AD is the modulation of α7 nicotinic receptors (nAChRs), important receptors in the hippocampus and prefrontal cortex for learning, memory, and executive function. Targeting only α7 nAChR, instead of all cholinergic receptors, as IAch drugs do, should reduce toxicity (Colovic et al., 2013; Yakel, 2013).

Both ABT-126, a selective α7 nicotinic receptor agonist, and ABT-089, an α4β2 neuronal nicotinic receptor partial agonist, showed no therapeutic effect (Lenz et al., 2015; Florian et al., 2016; Gault et al., 2016). ABT-126 was generally well tolerated but also induced agitation, constipation, diarrhea, fall and headache (Florian et al., 2016; Gault et al., 2016).

### Glutamatergic pathways

Glutamatergic neurotransmission related to N-methyl-d-aspartate (NMDA) function in cortical and hippocampal brain regions also plays a relevant role in AD pathogenesis. Memantine is an approved medication for this target. Activation of NMDA receptor signaling pathway produces secondary messengers, such as cyclic guanosine monophosphate (cGMP). Therefore, inhibition of phosphodiesterase type 9 (PDE9), which hydrolyzes cGMP, could increase cGMP levels and enhance cognition through long-term potentiation (LTP) (Reneerkens et al., 2009; Dubois et al., 2010). BI 409306 is a PDE9 inhibitor that was promising in rodents’ test, but no clinically meaningful changes were detected (Frölich et al., 2019).

### Glutaminyl cyclase

Glutaminyl cyclase (QC) plays a central role in synaptotoxic Aβ oligomer formation with pro-inflammatory potential. QC is an enzyme (glutaminyl peptide cyclotransferase, EC 2.3.2.5) that converts glutamate residue at position 3 of the N-terminus of truncated Aβ to AβpE3 peptide (Coimbra et al., 2019) and is involved in several pathological disorders, especially in AD (Vijayan and Zhang, 2019). Evidence have shown that AβpE3, a modified form of Aβ, may contribute to tau hyperphosphorylation (Mandler et al., 2014; Hennekens et al., 2015; Bayer, 2022). Indeed, AβpE3 is upstream in the neurotoxic amyloid cascade triggering neurodegeneration and influencing the severity of AD pathology (Pivtoraiko et al., 2015; Moro, et al., 2018). Since QC may catalyze the generation of cerebral AβpE3, its activity is overexpressed in AD brains, showing that QC inhibitors may be a good option for the development of AD-modifying strategies. The inhibitory activity of current inhibitors is mainly triggered by zinc-binding groups that coordinate zinc ion in the active site and other common features (Coimbra et al., 2019). Moreover, the inhibition of this enzyme also reduces the formation of mature CCL2, and thus suppresses neuroinflammation (Vijayan and Zhang, 2019). Although Scheltens et al. (2018) showed cognitive improvement with PQ912, some participants discontinued the trial due to high-dose toxicity. PQ912 targeted glutaminyl cyclase enzymes in patients with mild AD. The study was promising due to the functional improvement, inhibition of deterioration of synaptic activity, and reduction of neuroinflammation in patients.

### Neuroinflammation

Chronic brain inflammation is another pathological hallmark of AD. Neuroinflammation is initiated when glial cells are activated by neural environment or neuronal injury. Particularly, tumor necrosis factor-α (TNF-α) signaling plays a master role in this scenario and has been associated with neuronal excitotoxicity, synapse loss, and propagation of the inflammatory state. TNF-α signaling also exacerbates amyloidogenesis, including upregulation of BACE-1 expression (Song et al., 2021; Chen et al., 2022). Etanercept and thalidomide are TNF-α antagonists but none had therapeutic effects in clinical trials (Butchart et al., 2015; Decourt et al., 2017). Etanercept limitation is related to its pharmacokinetics; it is unable to cross the brain–blood barrier (Butchart et al., 2015). Thalidomide had poor tolerability (Decourt et al., 2017).

Some already approved drugs were also in evaluation for AD as an off-label strategy. A calcium channel blocker antihypertensive drug, nilvadipine, reduced amyloid production, increased cerebral blood flow, and has demonstrated anti-inflammatory and anti-tau activity in preclinical studies. These properties could be related to a blood pressure control or anti-amyloid mechanisms (Paris et al., 2004; Paris et al., 2014). However, clinical trial results do not suggest benefit of nilvadipine as a treatment to AD (Lawlor et al., 2018; Abdullah et al., 2020). Insulin also was evaluated due to its metabolic, mitochondrial, and protease activity, influencing clearance of Aβ peptide and phosphorylation of tau (Valla et al., 2010; Kellar and Craft, 2020). Intranasal glulisine, an insulin analog lacking potential olfactory toxicity due to zinc ingredient commonly found in insulin formulations, also failed in therapeutic effects (Rosenbloom et al., 2021). Oxidative stress is one of multiple factors contributing to AD pathogenesis. Monoamine oxidase B (MAO-B) enzymes are related to this mechanism in astrocytes due to oxidative deamination of neurotransmitters. A number of MAO-B inhibitors (MAO-Bi) have been studied for AD and Parkinson’s disease, such as sembragiline a selective MAO-Bi. Sembragiline demonstrated a good safety profile and potential effect on neuropsychiatric symptoms and behaviorally impaired (Nave et al., 2017).

Natural products were also studied. A marine derivative PKC epsilon activator, namely, bryostatin, increased synaptic numbers *via* synaptic growth factors but showed no efficacy in trial (Farlow et al., 2019). MLC901 (NeuroAiD II) contains extracts from nine herbal components, and triggered neurogenesis and neuroproliferation in rodents and human stem cell cultures due to activating ATP-dependent potassium channels (KATP) and modulating neuroinflammation. Clinically, MLC601 as monotherapy showed better tolerability and comparable efficacy to AChEIs in patients with mild to moderate AD, vascular dementia, and mild cognitive impairment (Chen et al., 2022). Sodium oligomannate (GV-971) is a marine-derived oligosaccharide that can modulate gut microbiota, reducing neuroinflammation in the brain as observed in animal models. GV-971 demonstrated significant efficacy in improving cognition and was safe and well-tolerated (Xiao et al., 2021).

SIRT-1 showed good results in phase two clinical trials (Turner, et al., 2015; Moussa et al., 2017). SIRT-1 is a sirtuin, a deacetylase protein regulated by NAD+/NADH activated by caloric restriction, which itself decreases age-dependent cognitive decline in animal models (Turner et al., 2015; Moussa et al., 2017). Resveratrol is a potent activator of SIRT-1 and helped to maintain blood–brain barrier (BBB) integrity by reducing oxidative stress, and inhibiting of NF-κB and matrix metalloproteinase-9 (MMP9) release (Lin et al., 2010; Wang et al., 2016; Espinoza et al., 2017).

In addition, resveratrol induced adaptive immune responses (Moussa et al., 2017). However, in phase 2 study, a reduction in brain volume was also found, and Turner et al. (2015) suggested lack of benefits. Moussa et al. (2017) showed that resveratrol significantly attenuated declined Aβ levels in CSF, and decreased cognitive and functional decline. The drug also reduced plasma levels of pro-inflammatory producers.

## Conclusion

In addition to all technology and information in AD pathogenesis, the most common outcome of AD new drug clinical trials was the lack of efficacy. However, those results may be limited by the disease stage of patients because earlier therapy has better performance in AD. Due to aducanumab recent FDA approval, many of the studies were using antibodies against Aβ, but they showed no noteworthy results. 5-HT6 antagonists, tau inhibitors, and nicotinic agonists’ data were also discouraging. However, anti-Aβ vaccine, BACE inhibitor, and anti-neuroinflammation drugs led to promising results with some drugs showing clinical improvements and no toxicity.
