# Supplementary figures and images for: Advances in Alzheimer’s disease’s pharmacological treatment
Source: Front Pharmacol. 2023 Jan 26;14:1101452. doi: 10.3389/fphar.2023.1101452 (PMC9933512; doi:10.3389/fphar.2023.1101452)

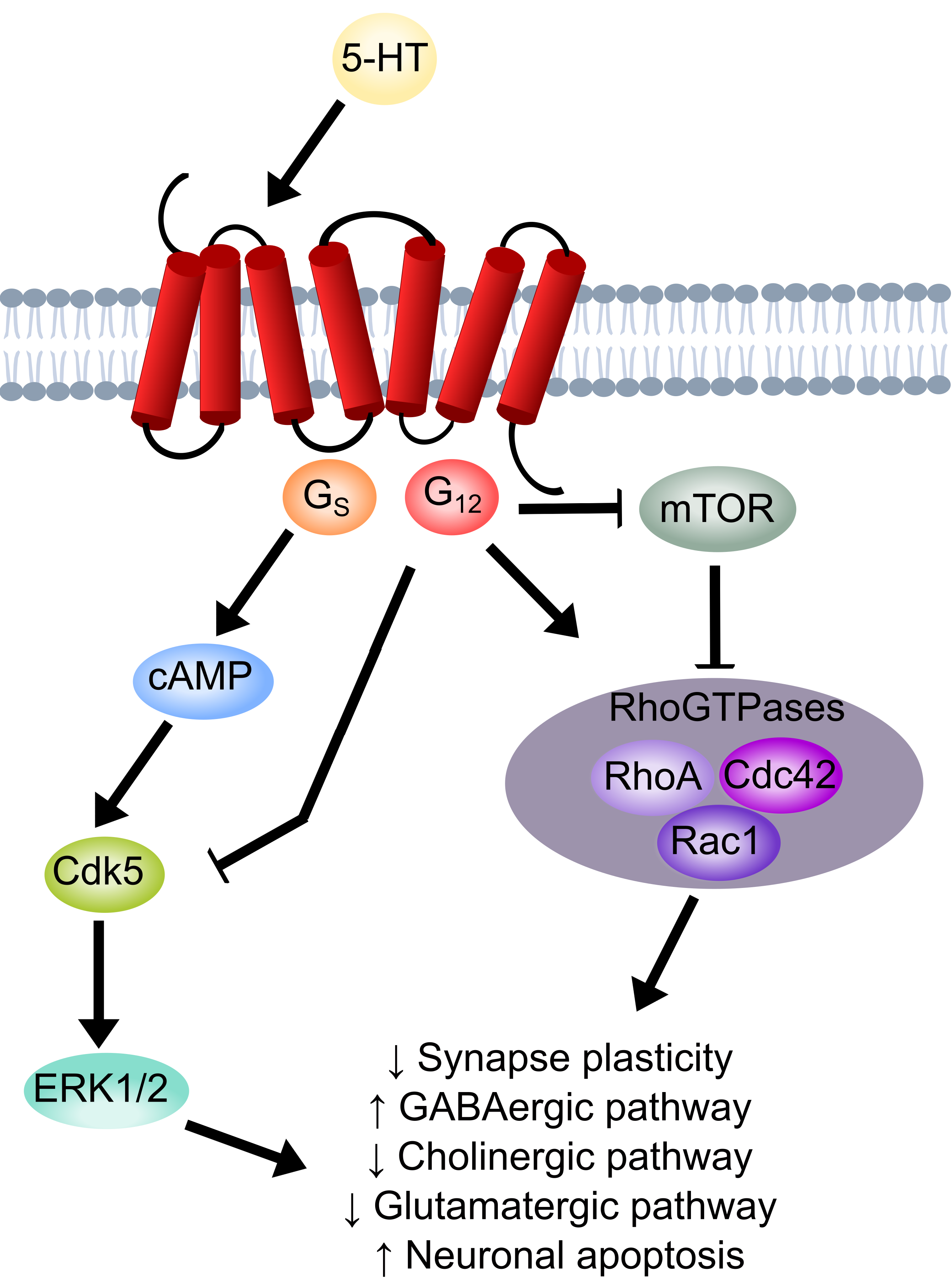

Supplement: Supplementary file 1 [file Image3.JPEG]

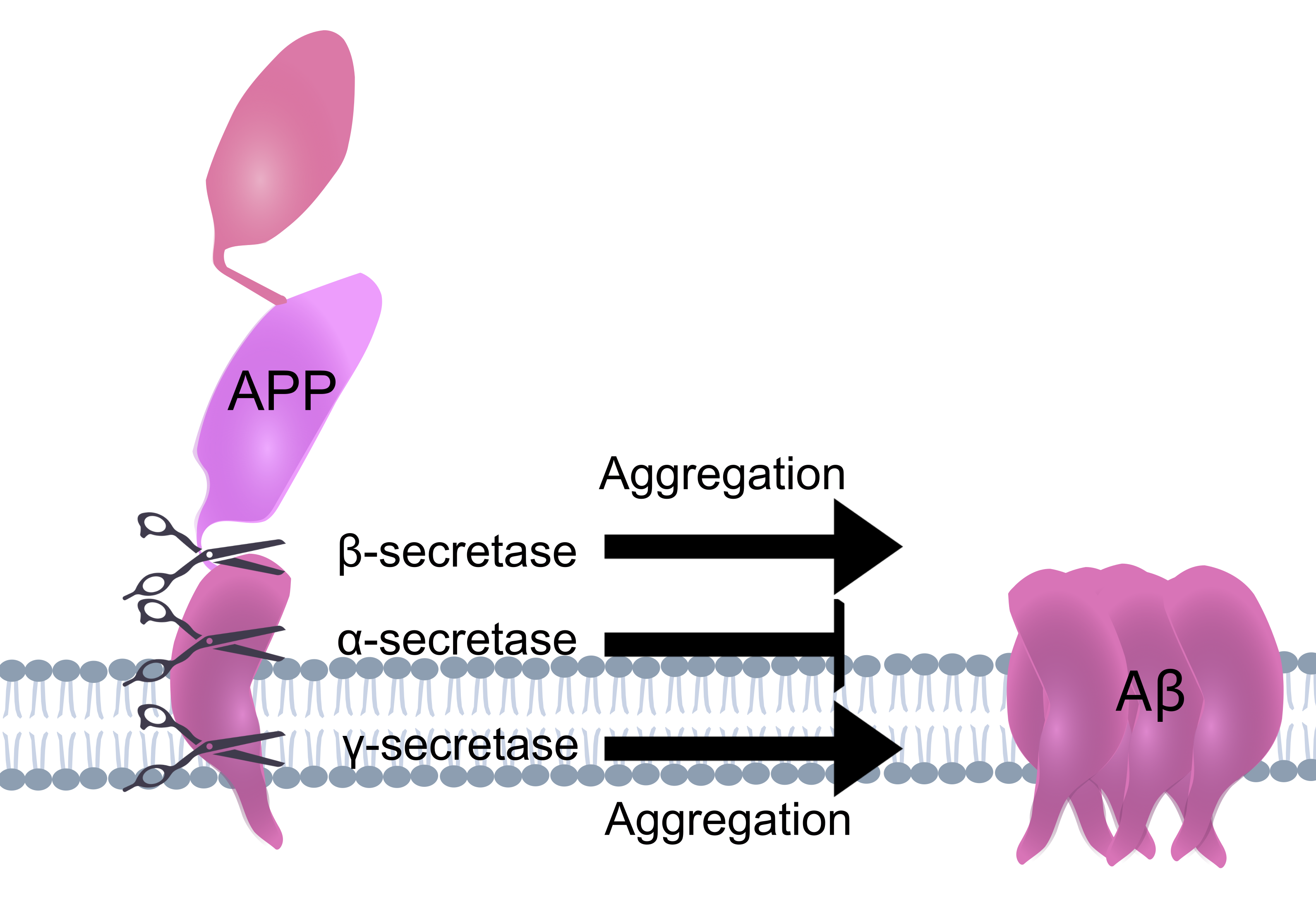

Supplement: Supplementary file 2 [file Image1.JPEG]

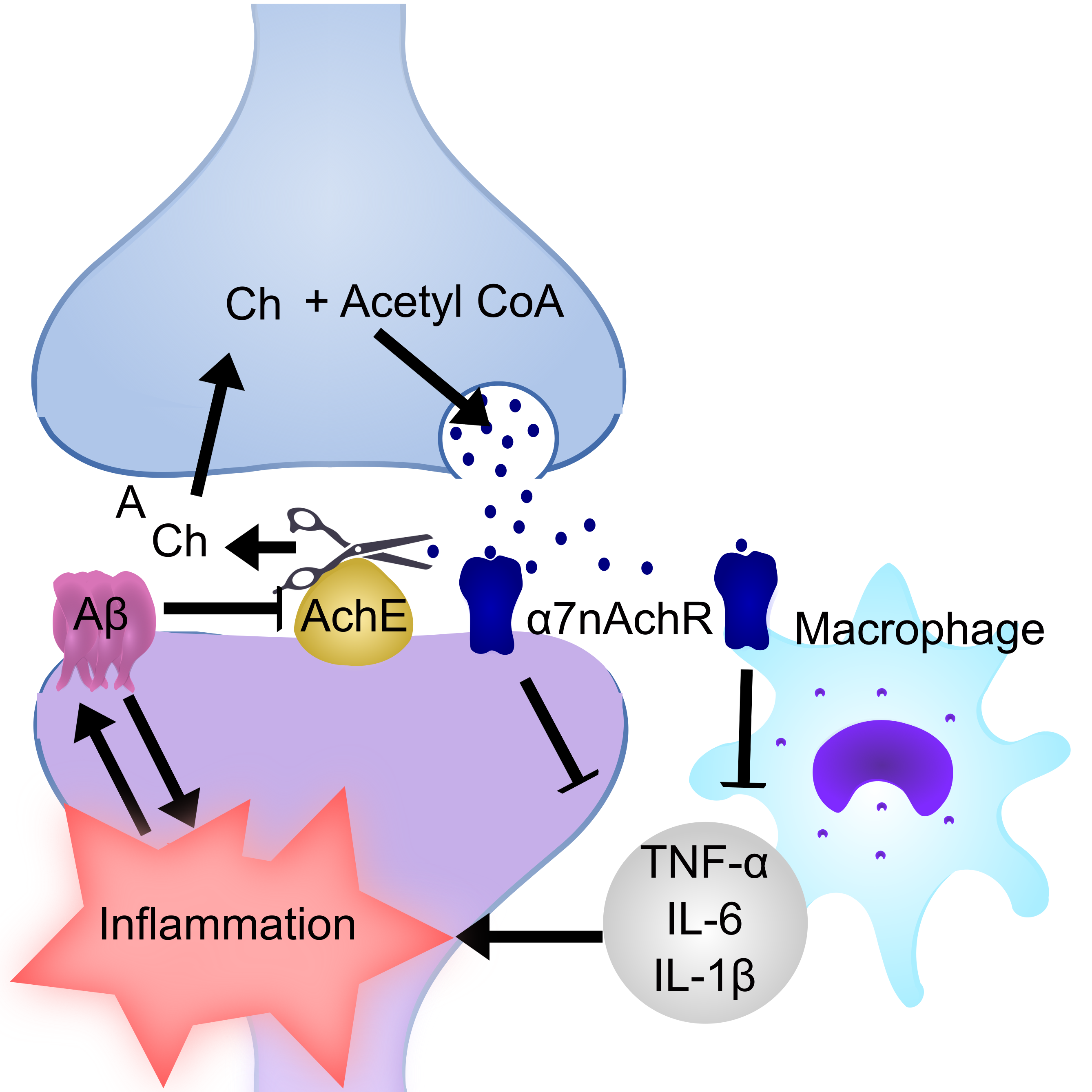

Supplement: Supplementary file 3 [file Image4.JPEG]

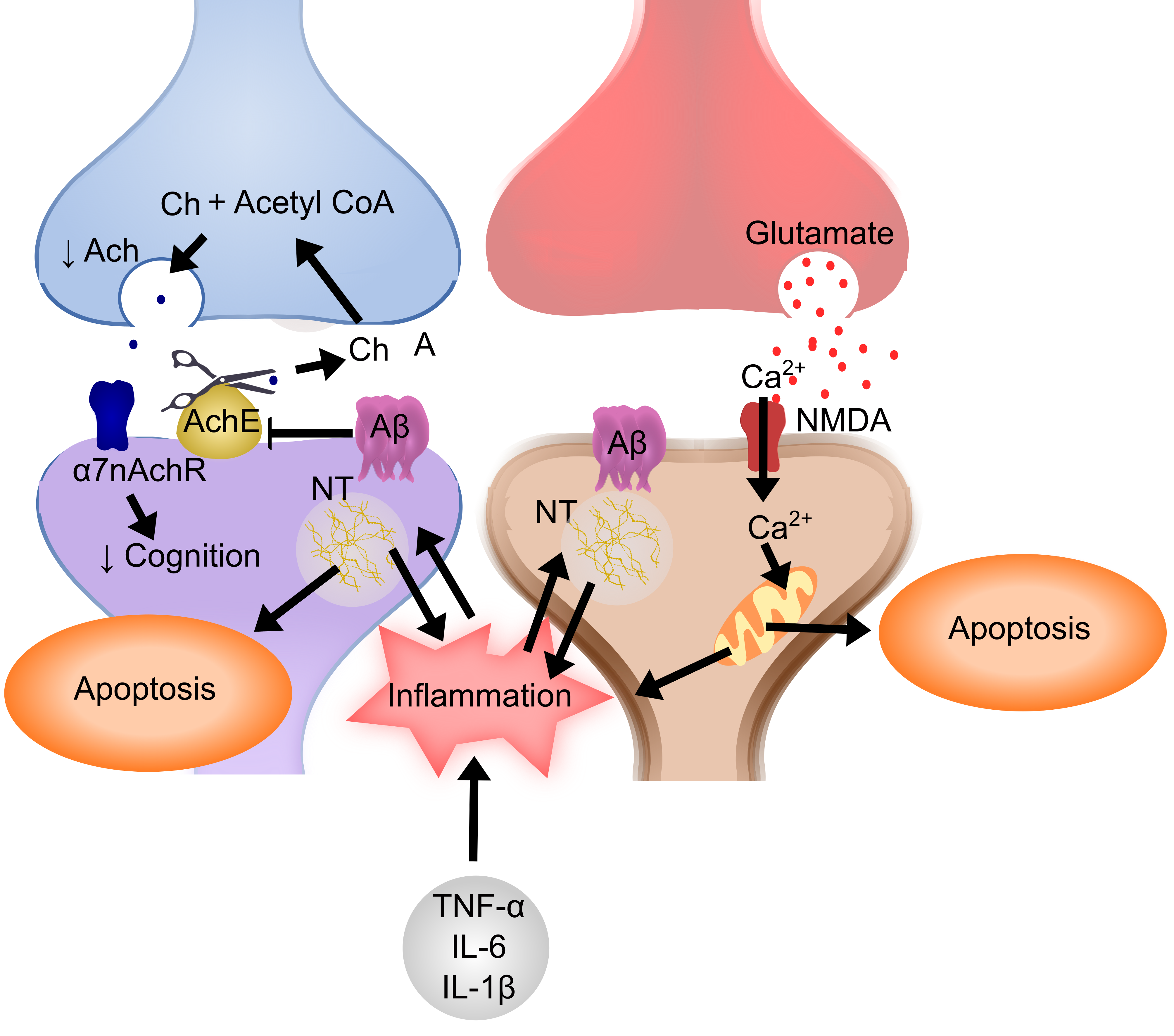

Supplement: Supplementary file 4 [file Image7.JPEG]

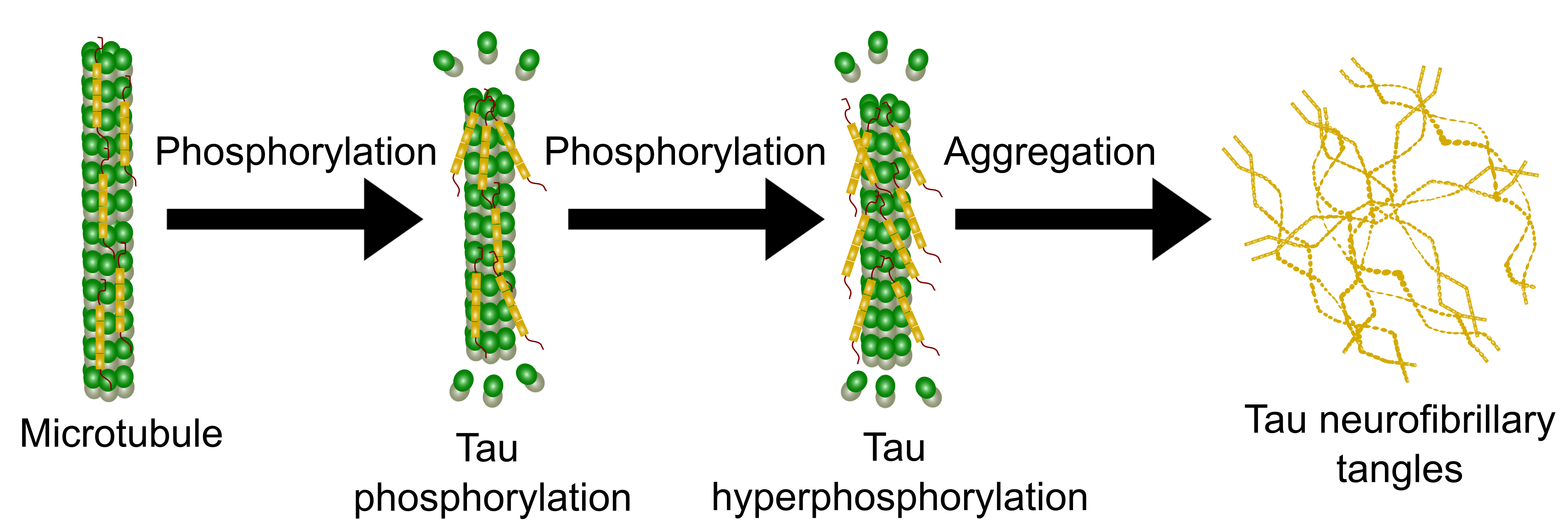

Supplement: Supplementary file 5 [file Image2.JPEG]

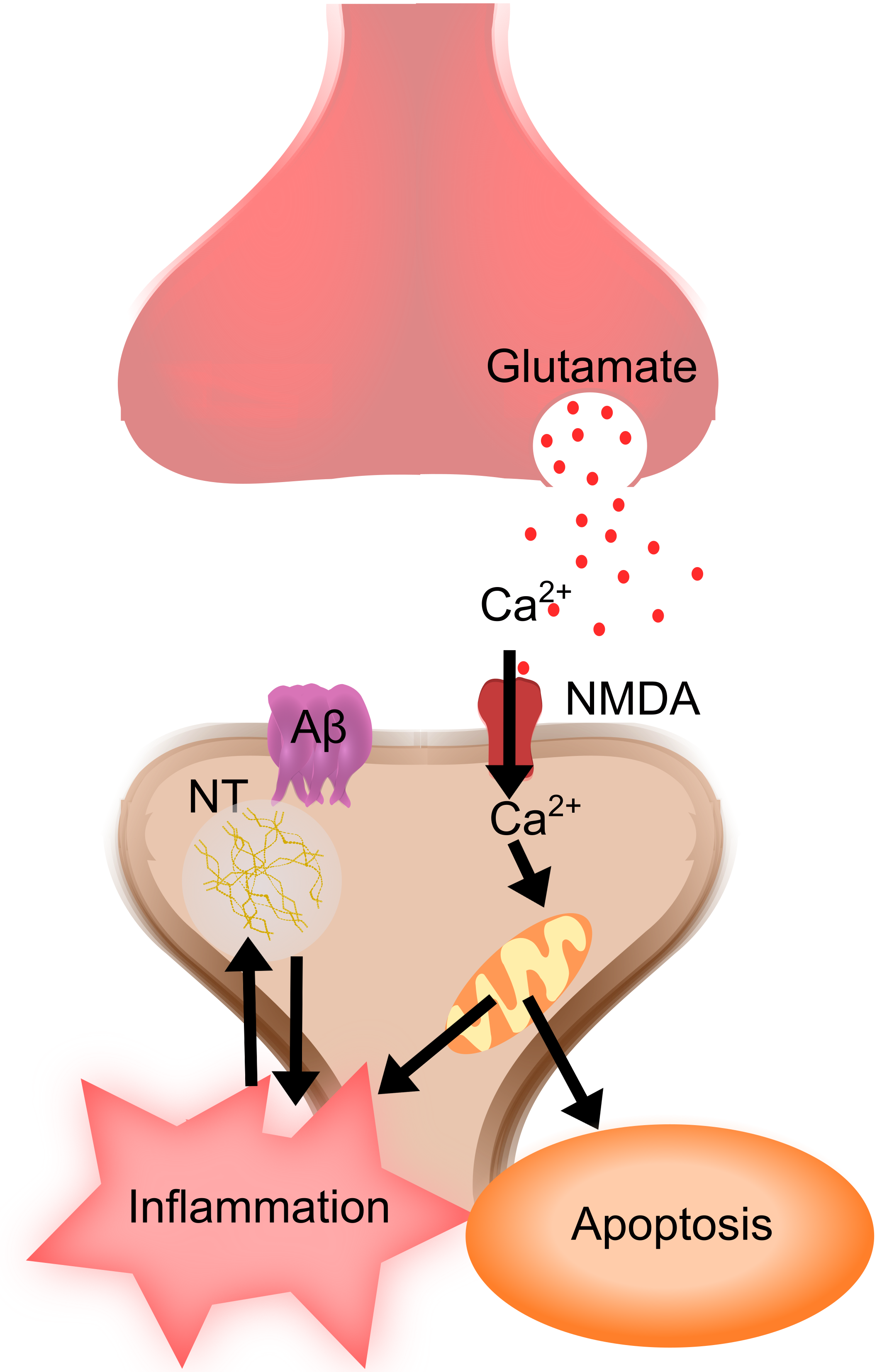

Supplement: Supplementary file 6 [file Image5.JPEG]

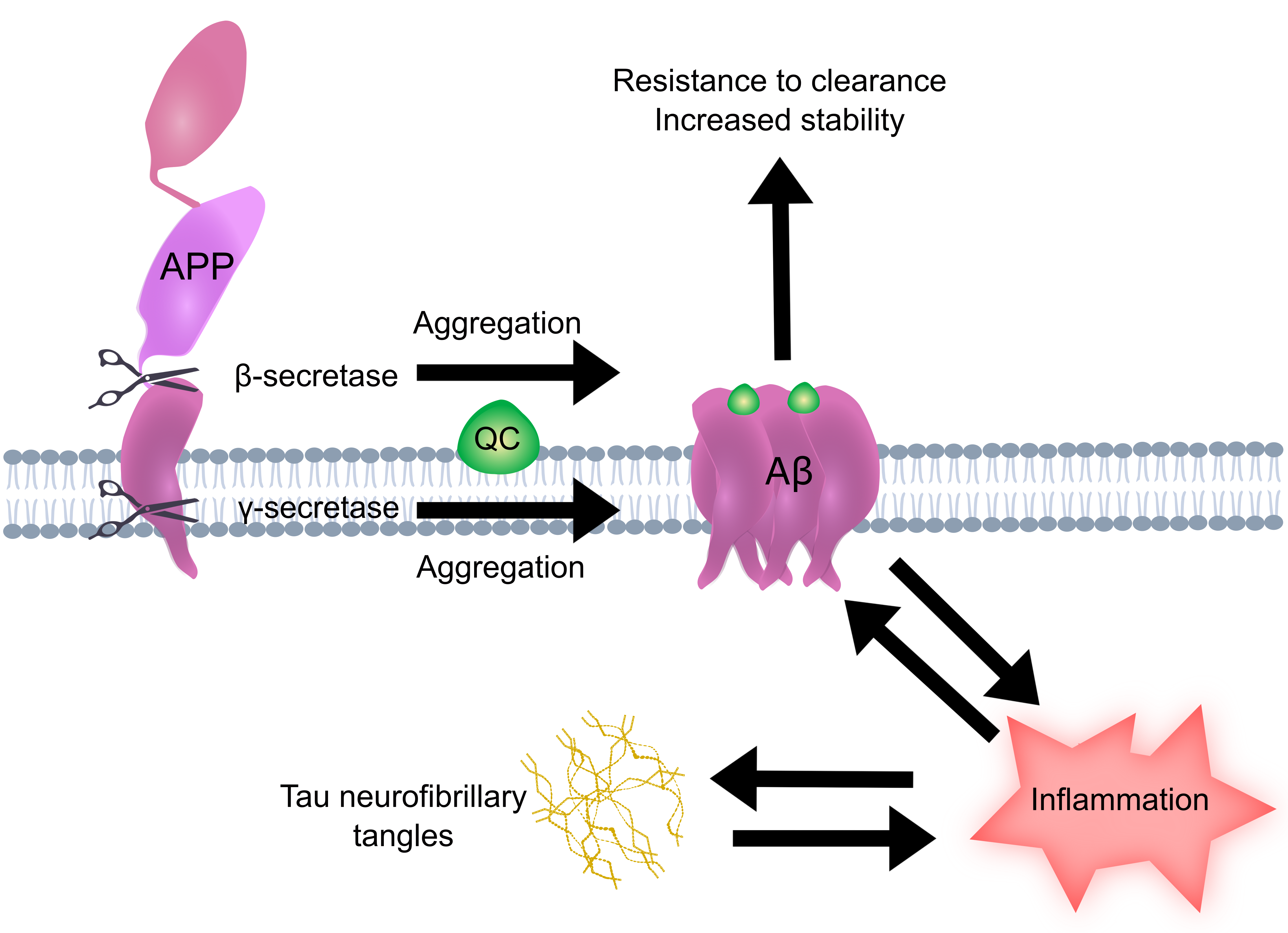

Supplement: Supplementary file 8 [file Image6.JPEG]
