# Supplementary material for: Advances in Alzheimer’s disease’s pharmacological treatment
Source: Front Pharmacol. 2023 Jan 26;14:1101452. doi: 10.3389/fphar.2023.1101452 (PMC9933512; doi:10.3389/fphar.2023.1101452)
Supplement: Supplementary file 7 [file DataSheet1.PDF]

Supplementary Material

Advances in Alzheimer's disease's pharmacological treatment

Carlos Elias Conti Filho†, Lairane Bridi Loss†, Clairton Marcolongo Pereira, Joamyr Victor Rossoni Junior, Rafael Mazioli Barcelos, Orlando Chiarelli Neto, Bruno Spalenza da Silva, Roberta Passamani Ambrosio, Fernanda Cristina de Abreu Quintela Castro, Sarah Fernandes Teixeira*, Nathana Jamille Mezzomo*

†These authors contributed equally to this work and share first authorship

*** Corresponding Authors:** sfteixeira@unesc.br and nathanajamillemezzomo@gmail.com

1 Supplementary Data

Most important targets nowadays to AD treatment are Aβ, tau, BACE-1, γ-secretase, cholinergic pathway, excitotocicity, glutaminyl cyclase and neuroinflammation. In the main text, we focused in good and bad outcomes of drug design pipelines. On the other hand, here were summarized the relation of these aimed targets to AD.

1.1 Aβ, tau, BACE-1 and γ-secretase

Senile plaques are Aβ proteins generated by amyloid precursor proteins (AAP) cleavage by β-secretases (BACE1) or γ-secretases, in both cases the product has hydrophobic parts and tend to aggregate. These proteins accumulation lead to neuronal apoptosis and inflammation. All individuals form Aβ, tough, as we age our immune system is less efficient in destroy it and we have more oxidative stress, leading to more misfolded proteins and inflammation. Physiological role of APP remains unknown, but it is usually cleaved by α-secretases and produces a hydrophilic product enable to form senile plaques (Lieblein et al., 2020; Madrasi et al., 2021; Prasanna and Jing, 2021; Han et al., 2022) (Supplementary Figure 1).

Tau deposition occurs in AD late stages due microtubule hyperphosphorylation, leading to tau aggregation into neurofibrillary tangles (NT) (Supplementary Figure 2). Microtubule is related to cell division, cell motility, intracellular transport and maintenance of cell shape. All these functions only work properly due to microtubule ability of polymerizate and depolymerizate, which is regulated by tau phosphorylation. NT also induce apoptosis and inflammation (Labus et al., 2021; Pereira et al., 2021; Zeng et al., 2021).

1.2 5-HT receptors

Recently 5-HT7R and 5-HT6R were highlighted in AD scenario due to it cognition, learning and memory roles, even though, their pathways are not entirely known (da Silva et al., 2021; Solas et al., 2021; Higa et al., 2022). Both receptor activates Gαs proteins, increasing cAMP and it activates protein kinase A (PKA) leading to phosphorylation of cyclin-dependent kinase 5 (Cdk5) and mitogen-activated protein kinase extracellular signal-regulated kinases 1/2 (ERK 1/2). By this mechanism, it decreases synaptic plasticity and cholinergic and glutamatergic pathways while it increases GABAergic pathways and neuronal apoptosis. Those receptors also interact with Gα12, stimulating Rho GTPases

and leading to the same results and also inhibits the mammalian target of rapamycin (mTOR) signalling (Liu et al., 2019; Chaumont-Dubel et al., 2020; Kusek et al., 2021) (Supplementary Figure 3). Therefore, developing 5-HT7R and 5-HT6R antagonists may be a solution for AD.

1.3 Cholinergic pathways

AD classic marks are reduction of acetylcholine and hippocampal shrink, which led to cholinergic hypothesis. Although this hypothesis is old, it is still evolving. Choline acetyltransferase (ChAT) syntheses acetycholine (ACh) from Acetyl-S-CoA (Acetyl CoA) and choline (Ch). In neuronal cells, ACh is stored in vesicles and is exocyted after a potential action, but other cells also can syntheses ACh, but it is released immediately. ACh binds to nicotinic acetylcholine receptors (nAChRs) or muscarinic acetylcholine receptors (mAChRs) on post-synaptic neurons or immune cells to induces its physiological roles. Membrane acetylcholinesterase (AChE) can degrade ACh into choline and acetic acid (A). Extracellular choline is transported into cells to be recycled (Recio-Barbero et al., 2021; Sabandal et al., 2022).

In particular, α7 nicotinic receptors are frequent in hippocampus and prefrontal cortex and are related to learning, memory and executive function. Moreover, these receptors in neurons and immune cells inhibit tumor necrosis factor α (TNF-α) and interleukins 6 and 1β (IL 6 and IL1β) activity, decreasing inflammation and Aβ development. Furthermore, Aβ production during AD progression also disrupt cholinergic pathway due to reduction of AChE activity and, thereafter, decrease of choline recycle. Aβ also trigger death of cholinergic neurons and boots inflammation (Gamage et al., 2020; Siddiqui et al., 2021; Shen et al., 2021) (Supplementary Figure 4).

1.4 Glutamatergic pathways

N-methyl-d-aspartate (NMDA) is a type of calcium ionotropic glutamate receptor involved in learning, memory and synaptic plasticity present in cortex and hippocampus. Although, overactivation of glutamatergic pathways through NMDA triggers calcium cell death mediated, called excitotoxicity (Hardingham, 2020; Dore et al., 2021; Zhang et al., 2021) (Supplementary Figure 5).

1.5 Glutaminyl cyclase

Glutaminyl cyclase (QC) is an enzyme present in humans in a soluble and a Golgi-resident form. It synthesis of one type Aβ oligomers from APP fragments, thus, it has a proinflammatory role and this scenario favours more misfolding proteins, such as Aβ and tau hyperphosphorylation (Coimbra et al., 2019; Bayer, 2022) (Supplementary Figure 6).

1.6 Neuroinflammation

As previously described in this text, chronic brain inflammation is intimately related to AD pathogenesis and progression. Mitochondrial dysfunction and cellular energy deficits increases as we age and promote inflammation, misfolding proteins accumulation and cell death. Systemic inflammation or neuronal injury activates glial cells and TNF-α signaling plays a master role in this scenario exacerbating amyloidogenesis by BACE-1 upregulation and potencializing excitotoxicity (Song et al., 2021; Chen et al., 2022) (Supplementary Figure 7).

2 Supplementary Figures and Tables

2.1 Supplementary Figures

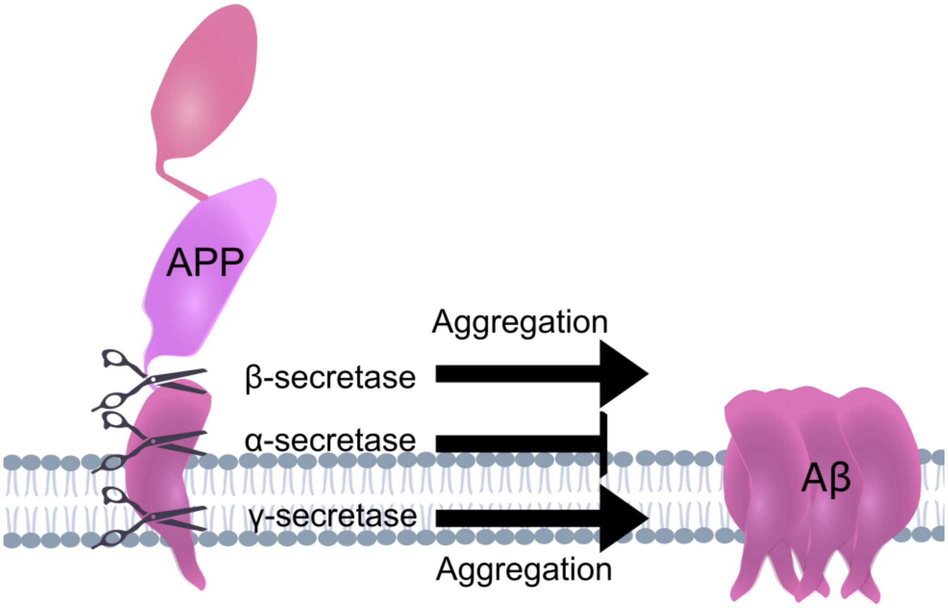

Supplementary Figure 1. Senile plaques formation pathway. Amyloid precursor proteins (APP) is a membrane protein that triggers Aβ formation through APP's fragments aggregation after by β-secretases (BACE1) or γ-secretases cleavage. APP is also an α-secretase substrate, but this reaction prevents senile plates development.

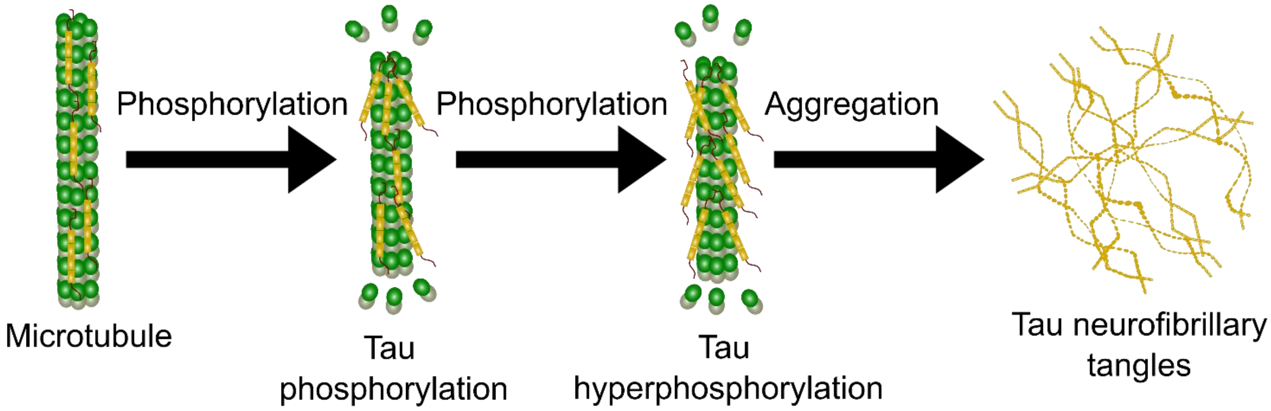

Supplementary Figure 2. Neurofibrillary tangles (NT) formation. NT are tau (in yellow) aggregates formatted by tau hyperphosphorylation.

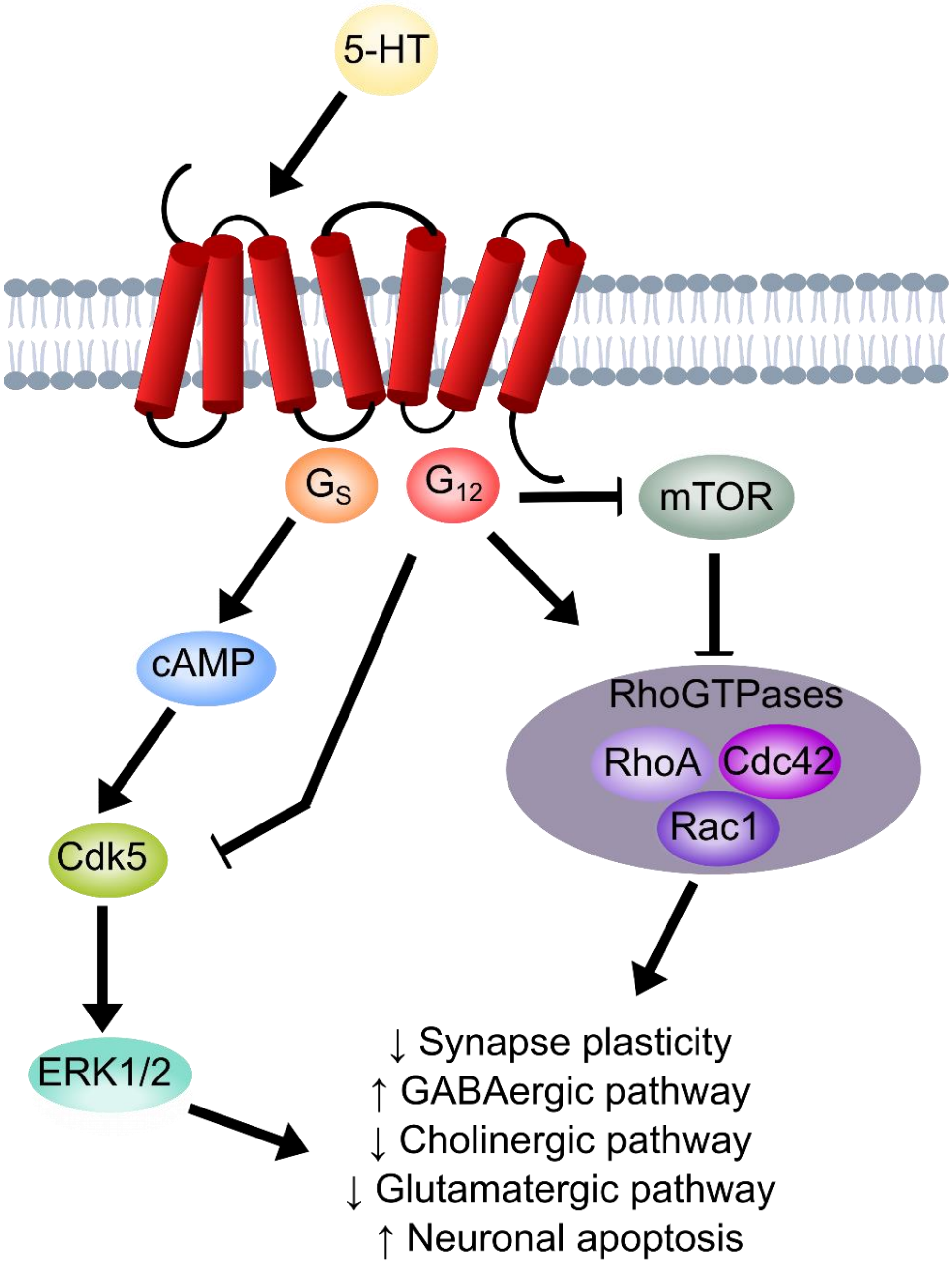

Supplementary Figure 3. Cognition decline driven by serotonin. Serotonin activates 5-HT7R and 5-HT6R, decreasing synapse plasticity, increasing neuronal apoptosis and disrupting neurotransmitters pathways through two different mechanisms. The first one involving Gαs, cAMP, PKA, Cdk5 and ERK 1/2. The other one is triggered by Gα12 and Rho GTPases.

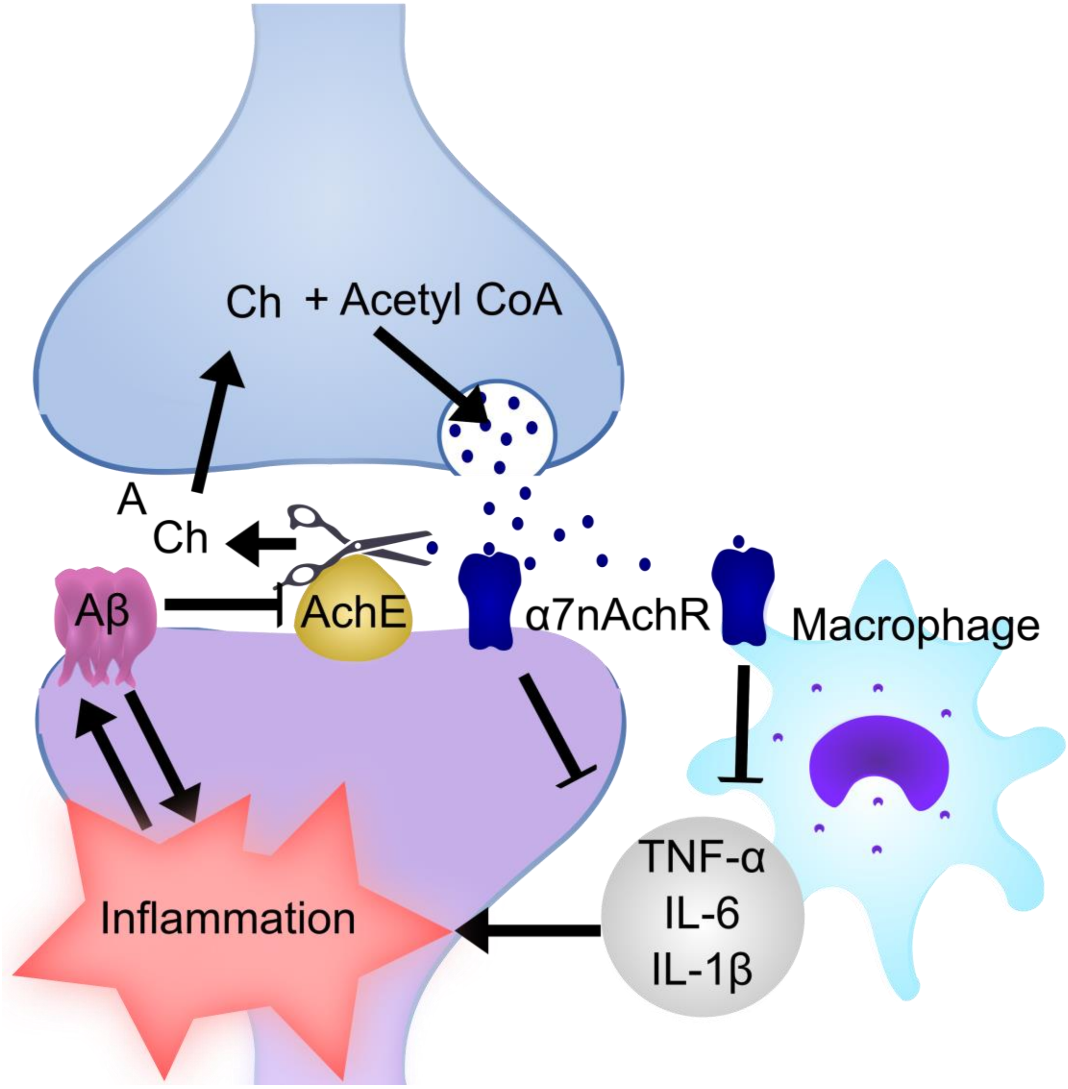

Supplementary Figure 4. Cholinergic hypothesis's mechanisms. Acetycholine (ACh) is an important neurotransmitter to cognition and is synthetized acetyl-S-CoA (Acetyl CoA) and choline (Ch) by choline acetyltransferase (ChAT). ACh binds to α7 nicotinic acetylcholine receptors (α7nAChRs) on post-synaptic neurons or immune cells to inhibit tumor necrosis factor α (TNF-α) and interleukins 6 and 1β (IL 6 and IL1β) activity, decreasing inflammation and Aβ development. Membrane acetylcholinesterase (AChE) can degrade ACh into choline and acetic acid (A). Extracellular choline is transported into cells to be recycled.

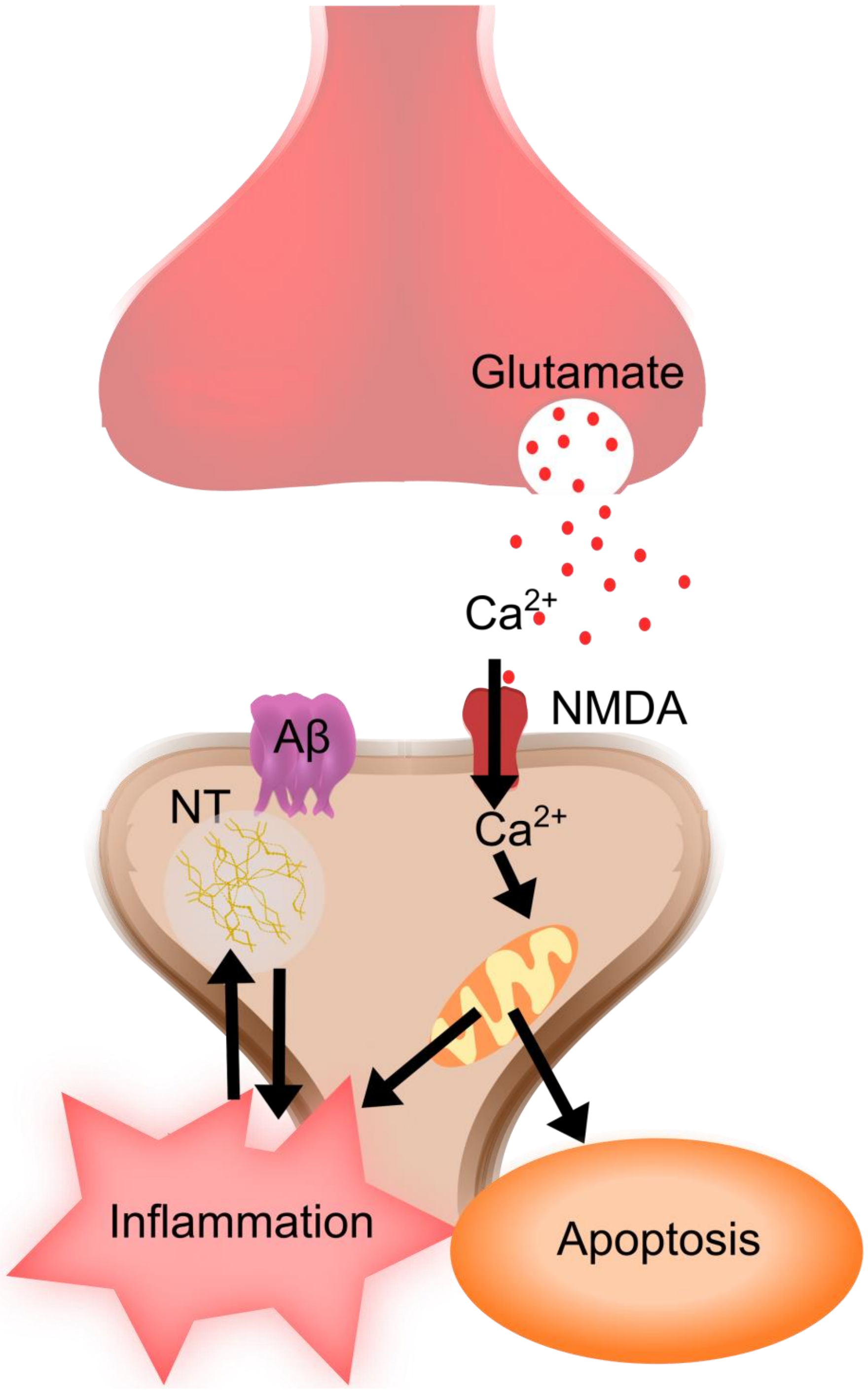

Supplementary Figure 5. AD relation to excitotoxicity. Excitotoxicity is triggered by N-methyl-d-aspartate (NMDA) glutamate receptor activation by excessive glutamate release. NMDA allows a calcium influx, which induces apoptosis and inflammation (increasing Aβ and neurofibrillary tangles, NT, formation).

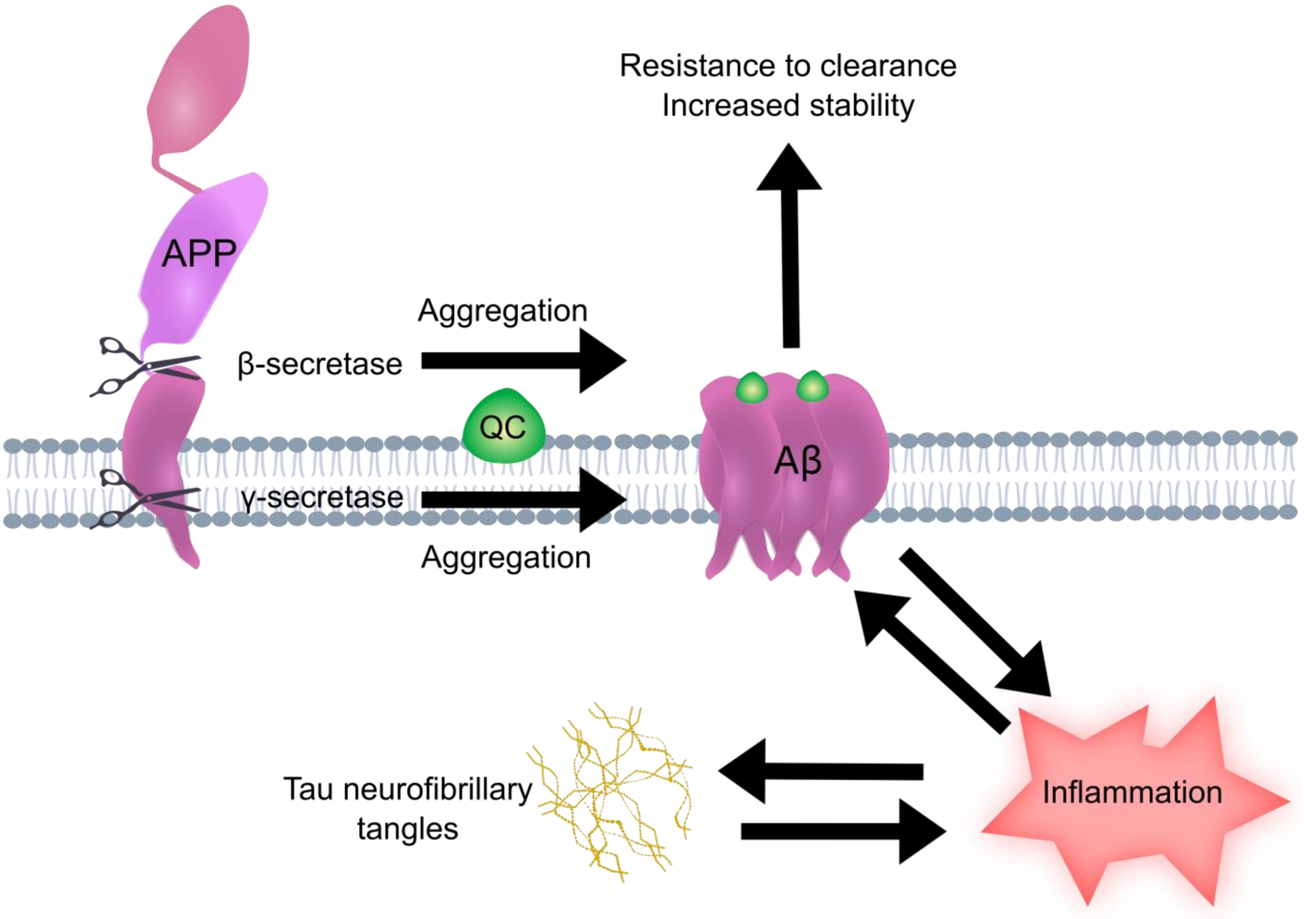

Supplementary Figure 6. Glutaminyl cyclase (QC) boots AD's pathogenesis. QC synthetizes one type Aβ oligomers more stable from APP fragments generating inflammation and more misfolding proteins.

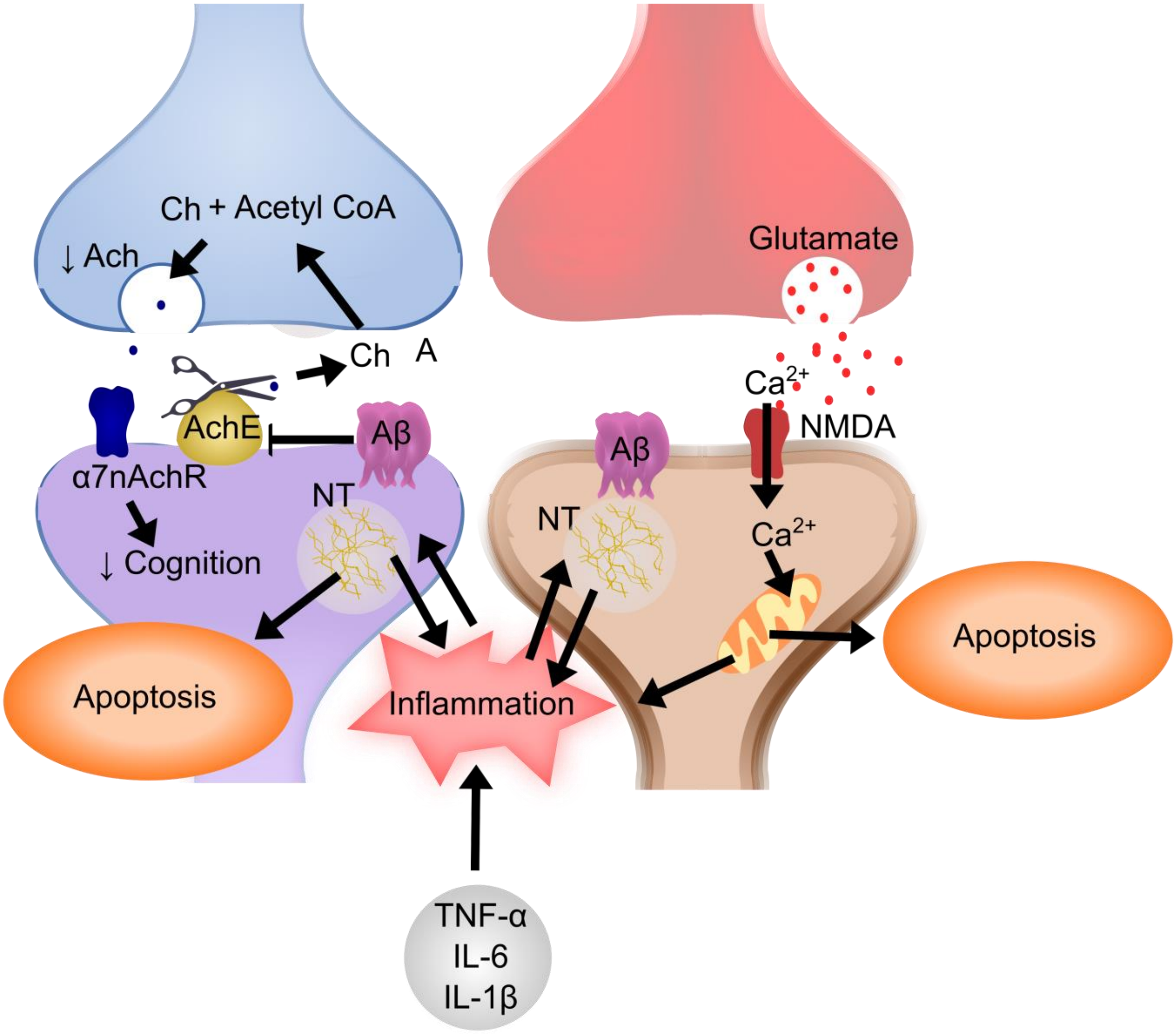

Supplementary Figure 7. Inflammation as a key to relate all mechanisms related to AD. Excitotoxicity, Aβ aggregation, neurofibrillary tangles (NT) formation, mitochondrial dysfunction and cellular energy deficits promote apoptosis and inflammation. Systemic inflammation or neuronal injury activates glial cells and tumor necrosis factor-α (TNF-α) signaling plays a master role in this scenario exacerbating amyloidogenesis and excitotoxicity.

2 References

Bayer, T.A. (2022). Pyroglutamate Aβ cascade as drug target in Alzheimer's disease. *Mol. Psychiatry*. 27, 1880-1885. doi: 10.1038/s41380-021-01409-2.

Chaumont-Dubel, S., Dupuy, V., Bockaert, J., Bécamel, C. and Marin, P. (2020). The 5-HT6 receptor interactome: New insight in receptor signaling and its impact on brain physiology and pathologies. *Neuropharmacology*. 172, 107839. doi: 10.1016/j.neuropharm.2019.107839.

Chen, C., Liao, J., Xia, Y., Liu, X., Jones, R., Haran, J., McCormick, B. et al. (2022). Gut microbiota regulate Alzheimer's disease pathologies and cognitive disorders via PUFA-associated neuroinflammation. *Gut*. 11, 2233-2252. doi: 10.1136/gutjnl-2021-326269.

Coimbra, J.R.M., Sobral, P.J.M., Santos, A.E., Moreira, P.I. and Salvador, J.A.R. (2019). An overview of glutaminyl cyclase inhibitors for Alzheimer's disease. *FutureMed. Chem.* 24, 3179-3194. doi: 10.4155/fmc-2019-0163.

da Silva, A.P., Chiari, L.P.A., Guimaraes, A.R., Honorio, K.M. and da Silva, A.B.F. (2021). Drug design of new 5-HT6R antagonists aided by artificial neural networks. *J. Mol. Graph. Model.* 104, 107844. doi: 10.1016/j.jmgm.2021.107844.

Dore, K., Carrico, Z., Alfonso, S., Marino, M., Koymans, K., Kessels, H.W. and Malinow, R. (2021). PSD-95 protects synapses from β-amyloid. *Cell Rep.* 9, 109194. doi: 10.1016/j.celrep.2021.109194.

Gamage, R., Wagnon, I., Rossetti, I., Childs, R., Niedermayer, G., Chesworth, R. and Gyengesi, E. (2020). Cholinergic Modulation of Glial Function During Aging and Chronic Neuroinflammation. *Front. Cell Neurosci.* 14, 577912. doi: 10.3389/fncel.2020.577912.

Han, C., Yang, Y., Guan, Q., Zhang, X., Shen H, Sheng Y. and Wang, J. (2020). New mechanism of nerve injury in Alzheimer's disease: β-amyloid-induced neuronal pyroptosis. *J. Cell. Mol. Med.* 14, 8078-8090. doi: 10.1111/jcmm.15439.

Hardingham, G.E. (2020). Targeting Synaptic NMDA Receptor Co-agonism as a Therapy for Alzheimer's Disease? *Cell Metab.* 3, 439-440. doi: 10.1016/j.cmet.2020.02.009.

Higa, G.S.V., Francis-Oliveira, J., Carlos-Lima, E., Tamais, A.M., Borges, F.D.S., Kihara, A.H., Shieh, I.C. et al. (2022). 5-HT-dependent synaptic plasticity of the prefrontal cortex in postnatal development. *Sci. Rep.*1, 21015. doi: 10.1038/s41598-022-23767-9.

Kusek, M., Siwiec, M., Sowa, J.E., Bobula, B., Bilecki, W., Ciurej, I., Kaczmarczyk, M. et al. (2021). 5-HT7 receptors enhance inhibitory synaptic input to principal neurons in the mouse basal amygdala. *Neuropharmacology.* 198, 108779. doi: 10.1016/j.neuropharm.2021.108779.

Labus, J., Röhrs, K.F., Ackmann, J., Varbanov, H., Müller, F.E., Jia, S., Jahreis, K., et al. (2021). Amelioration of Tau pathology and memory deficits by targeting 5-HT7 receptor. *Prog. Neurobiol.* 197,101900. doi: 10.1016/j.pneurobio.2020.101900.

Lieblein, T., Zangl, R., Martin, J., Hoffmann, J., Hutchison, M.J., Stark, T., Stirnal, E., Schrader,T. et al. (2020). Structural rearrangement of amyloid-β upon inhibitor binding suppresses formation of Alzheimer's disease related oligomers. *Elife*. 9, e59306. doi: 10.7554/eLife.59306.

Liu, P., Yin, Y.L., Wang, T., Hou, L., Wang, X.X., Wang, M., Zhao, G.G. et al. (2019). Ligand-induced activation of ERK1/2 signaling by constitutively active Gs-coupled 5-HT receptors. *Acta. Pharmacol. Sin.* 9, 1157-1167. doi: 10.1038/s41401-018-0204-6.

Madrasi, K., Das, R., Mohmmadabdul, H., Lin, L., Hyman, B.T., Lauffenburger, D.A. and Albers, M.W. (2021). Systematic in silico analysis of clinically tested drugs for reducing amyloid-beta

plaque accumulation in Alzheimer's disease. *Alzheimers Dement.* 9, 1487-1498. doi: 10.1002/alz.12312.

Pereira, J.B., Janelidze, S., Ossenkoppele, R., Kvartsberg, H., Brinkmalm, A., Mattsson-Carlgren, N., Stomrud, E. et al. (2021). Untangling the association of amyloid-β and tau with synaptic and axonal loss in Alzheimer's disease. *Brain.* 1, 310-324. doi: 10.1093/brain/awaa395.

Prasanna, G. and Jing, P. (2021). Self-assembly of N-terminal Alzheimer's β-amyloid and its inhibition. *Biochem. Biophys. Res. Commun.* 534, 950-956. doi: 10.1016/j.bbrc.2020.10.065.

Recio-Barbero, M., Segarra, R., Zabala, A., González-Fraile, E., González-Pinto, A. and Ballesteros, J. (2021). Cognitive Enhancers in Schizophrenia: A Systematic Review and Meta-Analysis of Alpha-7 Nicotinic Acetylcholine Receptor Agonists for Cognitive Deficits and Negative Symptoms. *Front. Psychiatry*. 12, 631589. doi: 10.3389/fpsyt.2021.631589.

Sabandal, P.R., Saldes, E.B. and Han, K.A. (2022). Acetylcholine deficit causes dysfunctional inhibitory control in an aging-dependent manner. *Sci. Rep.* 1, 20903. doi: 10.1038/s41598-022-25402-z.

Shen, H., Meng, Y., Liu, D., Qin, Z., Huang, H., Pan, L., Wang, W. and Kang, J. (2021). α7 Nicotinic Acetylcholine Receptor Agonist PNU-282987 Ameliorates Cognitive Impairment Induced by Chronic Intermittent Hypoxia. *Nat. Sci. Sleep*. 13, 579-590. doi: 10.2147/NSS.S296701.

Siddiqui, A., Shah, Z., Jahan, R.N., Othman, I. and Kumari, Y. (2021). Mechanistic role of boswellic acids in Alzheimer's disease: Emphasis on anti-inflammatory properties. *Biomed. Pharmacother.* 144, 112250. doi: 10.1016/j.biopha.2021.112250.

Solas, M., Van Dam, D., Janssens, J., Ocariz, U., Vermeiren, Y., De Deyn, P.P. and Ramirez, M.J. (2021). 5-HT7 receptors in Alzheimer's disease. *Neurochem. Int.* 150, 105185. doi: 10.1016/j.neuint.2021.105185.

Song, T., Song, X., Zhu, C., Patrick, R., Skurla, M., Santangelo, I., Green, M. et al. (2021). Mitochondrial dysfunction, oxidative stress, neuroinflammation, and metabolic alterations in the progression of Alzheimer's disease: A meta-analysis of in vivo magnetic resonance spectroscopy studies. *Ageing Res. Rev*. 72, 101503. doi: 10.1016/j.arr.2021.101503.

Zhang, Z.H., Chen, C., Jia, S.Z., Cao, X.C., Liu, M., Tan, J., Hoffmann, P.R. et al. (2021). Selenium Restores Synaptic Deficits by Modulating NMDA Receptors and Selenoprotein K in an Alzheimer's Disease Model. *Antioxid. Redox Signal.* 11, 863-884. doi: 10.1089/ars.2019.7990.

Zeng, L., Jiang, H., Ashraf, G.M., Liu, J., Wang, L., Zhao, K., Liu, M. et al. (2021). Implications of miR-148a-3p/p35/PTEN signaling in tau hyperphosphorylation and autoregulatory feedforward of Akt/CREB in Alzheimer's disease. *Mol. Ther. Nucleic Acids*. 27, 256-275. doi: 10.1016/j.omtn.2021.11.019.
